# Mandibular metastasis of primary extrahepatic biliary carcinoma: Case report

**DOI:** 10.1016/j.ijscr.2022.107498

**Published:** 2022-08-10

**Authors:** Alessandra Kuhn Dall'Magro, Letícia Copatti Dogenski, Patrícia Bade, Larissa Cunha Cé, Eduardo Dall'Magro, João Paulo De Carli

**Affiliations:** a Department of Oral and Maxillofacial Surgery, São Vicente de Paulo Hospital, Passo Fundo, RS, Brazil; b Postgraduate Program in Odontology, University of Passo Fundo, Passo Fundo, RS, Brazil; c Graduation in Odontology, University of Passo Fundo, Passo Fundo, RS, Brazil; d Department of Dental Prothesis, University of Passo Fundo, Passo Fundo, RS, Brazil; e Department of Implantology and Dental Prosthesis, University of Passo Fundo, Passo Fundo, RS, Brazil

**Keywords:** Case report, Cholangiocarcinoma, Extrahepatic bile ducts, Mandibular metastasis

## Abstract

**Introduction and importance:**

Cholangiocarcinoma is a malignant neoplasm with a difficult diagnosis and unfavorable prognosis that can appear anywhere in the biliary tree. Anatomically, it can be classified as intra- or extrahepatic, the latter being the most common primary malignancy of the biliary tract. Although bone tissue is the third most common target for metastases in general, metastatic lesions from cholangiocarcinomas are uncommon in the head and neck.

**Case presentation:**

A 33-year-old female patient undergoing cancer treatment for cholangiocarcinoma was referred to the Oral and Maxillofacial Surgery and Traumatology Service (CTBMF) of the São Vicente de Paulo Hospital in Passo Fundo, Rio Grande do Sul, Brazil, for interdisciplinary follow-up. She presented trismus, dysphagia, pain, necrosis and drainage in the mental region, and an increased volume on the right side of the mandibular body in the region of teeth 47 and 48. An incisional biopsy was performed in this bulging region. The histopathological diagnosis was poorly differentiated carcinoma. The patient was followed-up weekly by the CTBMF team, receiving analgesia and palliative treatment for the extra- and intraoral lesions, controlling opportunistic infections, and improving food intake.

**Discussion:**

Metastatic cholangiocarcinomas in the mandible are rare, hence their diagnosis is often late, which may disfavor the prognosis of these conditions.

**Conclusion:**

Carefully examining patients with mandibular bone lesions is strongly suggested, especially patients with known or previous concomitant malignancy.

## Highlights

• Diagnosis of metastatic carcinoma can be challenging, mimicking other diseases.
• Biopsy and immunohistochemistry are essential for diagnosis of metastatic carcinoma.
• The dentist plays a fundamental role in diagnosis of metastatic carcinoma.
• Metastatic cholangiocarcinomas in the mandible are rare.

## 1 Introduction

Cholangiocarcinoma (CCA) is an uncommon and aggressive adenocarcinoma originating from cholangiocytes, which are epithelial cells that line the bile ducts, thus constituting a heterogeneous group of malignant diseases that can appear anywhere in the biliary tree [1], [2], [3]. The CCA is the second most common hepatic malignancy after hepatocellular carcinoma (HCC) [4]. This condition is difficult to diagnose and has an unfavorable prognosis, with survival lower than 5 % of cases in five years due to the tendency to insidious progression [1].

Based on its anatomical location relative to second-order bile ducts, CCA is classified as intrahepatic (IH) or extrahepatic (EH) [5]. Intrahepatic cholangiocarcinoma (IHCCA) appears in the peripheral bile ducts within the liver parenchyma. Extrahepatic cholangiocarcinoma (EHCCA) can involve the confluence of the bile duct in the liver hilum, being categorized as perihilar, or affect the lower or middle half of the bile duct, therefore called distal [6]. The EHCCA comprises approximately 75 % of all cases and is the most common primary malignancy of the biliary tract [2].

The EHCCA usually shows signs and symptoms of biliary obstruction (painless jaundice, itching, light stools, and dark urine) [5]. The early diagnosis, however, remains a challenge due to its silent clinical character, considering that most patients with the disease at an early stage are asymptomatic [4]. This delay in the diagnosis of EHCCA may contribute to the occurrence of metastases, which are the main cause of cancer lethality. These disseminated tumor cells travel to distant areas where they overflow from the circulation and enter the target tissue [7], [8].

Presenting a rich blood supply and a specific growth-promoting microenvironment, bone tissue is the third most common target for metastasis in general, after the liver and lungs [9]. However, metastatic tumors of the oral cavity are not common, representing only about 1 % of oral tumors [10]. This metastatic site has clinical importance because it may indicate a yet unknown cancer, generalization of an already diagnosed cancer, or recurrence of disease.

Considering that metastatic CCAs in the mandible are rare because the spread to other vital organs before the mandible can result in a low survival rate [1], the present report addresses the case of a young patient with mandibular metastasis of an extrahepatic bile duct carcinoma, treated in a public Hospital in Brazil. As far as we know, this is the only report in the literature of a case like this. Thus, this article aims to contribute to education by reporting its treatment and other relevant aspects of this rare neoplasm with an unfavorable prognosis.

## 2 Presentation of case

A free and informed consent form was obtained from the patient, allowing the publication of the case. All applicable standards regarding the ethics of experimentation and research integrity have been followed. This study was reported following the SCARE criteria [11].

**Fig. 1:**
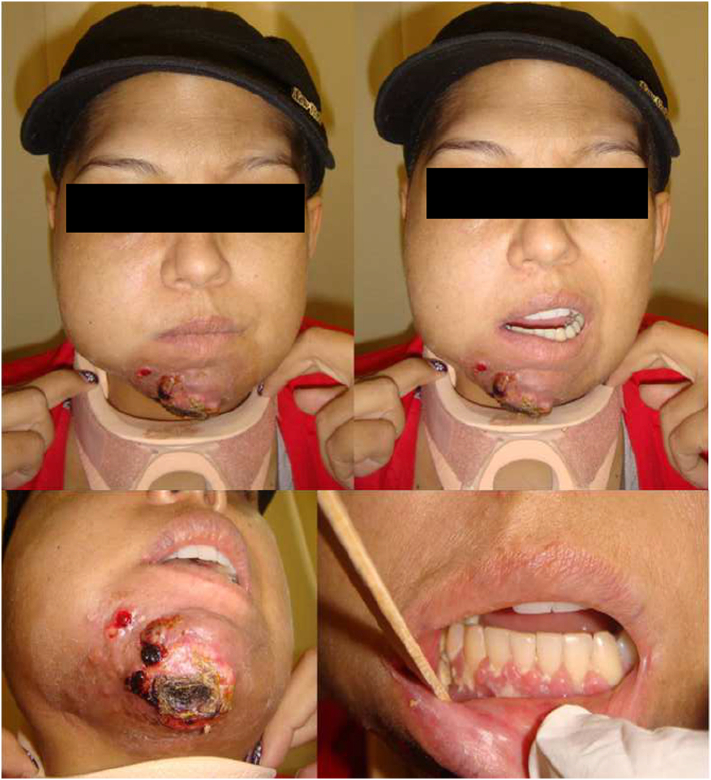
Clinical aspect of the edema on the right side of the face, drainage of the mental region, and trismus.

A 33-year-old female patient, student, undergoing cancer treatment for cholangiocarcinoma was referred to the Oral and Maxillofacial Surgery and Traumatology Service (CTBMF) of the São Vicente de Paulo Hospital in Passo Fundo, Rio Grande do Sul, Brazil, for interdisciplinary follow-up. She presented trismus, dysphagia, pain, necrosis and drainage in the mental region, and an increased volume on the right side of the mandibular body close to teeth 47 and 48 (Fig. 1). The patient had no drug history, family history including any relevant genetic information, and psychosocial history.

**Fig. 2:**
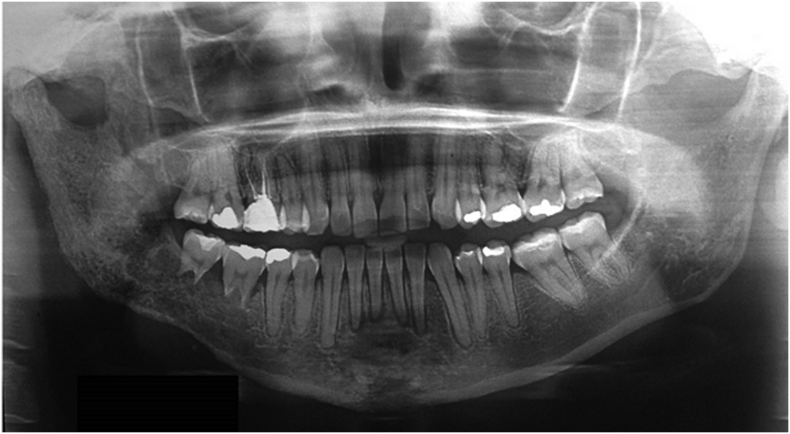
General radiography showing extensive root resorption, lysis of the trabecular bone, and diffuse cortical bone.

**Fig. 3:**
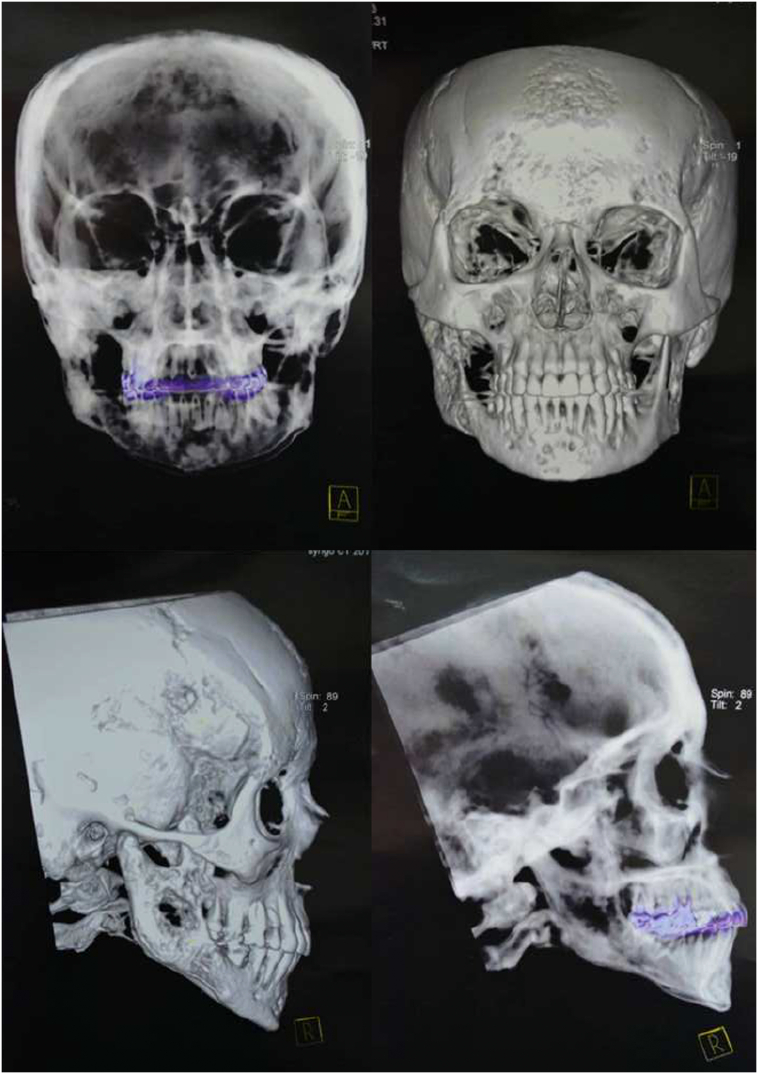
Computed tomography showing hypodense areas in the mandibular bone, skull, and cervical vertebra.

The analysis of the panoramic radiography showed extensive root resorption, lysis of the trabecular bone, and diffuse cortical bone on the posterior right side of the mandible (Fig. 2). A computed tomography scan revealed hypodense areas in the frontal and mandibular bones, skull, and cervical vertebrae (Fig. 3). Given the picture described, an incisional biopsy of the site was performed, which histopathological diagnosis was poorly differentiated carcinoma. Incisional biopsy was performed under local anesthesia with mepivacaine (1:100,000) in an outpatient setting, by a specialist in Oral and Maxillofacial Surgery and Traumatology, without postoperative interventions.

**Fig. 4:**
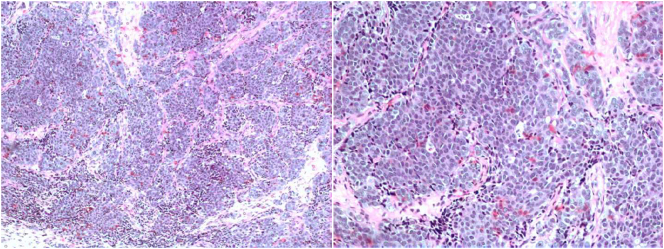
Histopathological aspect of poorly differentiated carcinoma showing malignant epithelial cells (100×) and condensed chromatin on the periphery of the nuclear membrane, grouped into solid cords, and bounded by connective septa of variable thickness (200×).

Microscopically, the neoplastic tissue that stood out consisted of rounded nuclear glands, irregularly distributed chromatin, and scarce and eosinophilic cytoplasm. Cells with condensed chromatin on the periphery of the nuclear membrane were frequent, grouping into solid strands, bounded by connective septa of variable thickness (Fig. 4). Additionally, fragments of the ascending mandibular ramus, extracted by frozen section biopsy on the right side, were diagnosed as a round cell tumor.

For a better classification of the tumor, an immunohistochemical study was performed with the avidin-biotin method, indirect immunoperoxidase with diaminobenzidine, and antigen recovery induced by heat or protease in a paraffin block. The primary antibodies used were cytokeratin, Clones AE1/AE3, acidic and basic cytokeratin marker at the origin of epithelial cells; cytokeratin 7 (CK7), Clone OV-TL12/30, usually reactive with the stratified squamous epithelium but also reacting with the transitional epithelium of the urinary system; cytokeratin 5/6, Clones D5/16B4, non-keratinized epithelium and epidermis marker; p63, clone 4A4, anti-human p63 protein antibody; CEA (CD66e), Clone 12-140-10, carcinoembryonic antigen marker; and BRST-2 (GCDFP-15), Clone 23A3, which identifies breast carcinoma (primary site or metastases) and extramammary Paget's disease.

**Fig. 5:**
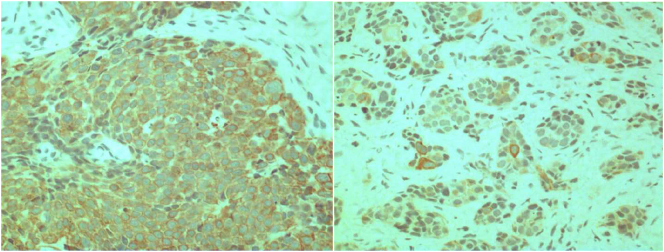
Immunohistochemistry with pan-cytokeratin antibody and CK7 antibody (200×).

The microscopic immunohistochemistry examination revealed neoplastic cells positive for anti-cytokeratin and anti-CK7 (Fig. 5). There was no positivity for anti-CK5/6, anti-p63, anti-CEA, and anti-BRST-2 antibodies, concluding the diagnosis of poorly differentiated carcinoma.

The patient's treatment for cholanginocarcinoma consisted of radio and chemotherapy with Fluoruracil 500 mg/m^2^ D1 to D3 bolus in the 1st and 5th week of radiotherapy, applied at a dose of 45 Gy in fractions of 180 cGy + boost of 5.4–9.0 Gy (total dose 54 Gy), which were well tolerated by the patient. After the diagnosis of mandibular metastasis, the patient was followed-up weekly by the CTBMF team, receiving analgesia and palliative treatment for the extra- and intraoral lesions, controlling opportunistic infections, and improving food intake.

The evolution occurred with significant impairment of mandibular bones and soft tissues, with increased swelling, pain, and drainage in the mental region. Moreover, there was an involvement of the cervical vertebral skeleton, concomitantly fractured and immobilized. At that point, radio- and chemotherapy were intensified. Then, the patient did not attend outpatient consultations and died 10 months after the initial diagnosis.

## 3 Discussion

The process of metastatic dissemination is complex and involves the individual characteristics of each tumor and qualities related to the host's response. Hence, there is a wide variety among tumor types regarding the frequency of metastases and their distribution in distant organs [12]. The oral cavity is a very uncommon site of metastasis and is usually evidence of a disseminated disease, which real incidence is difficult to determine precisely because an unknown number of patients die without investigating oral metastasis. The known cases of maxillofacial metastases involve only patients who have had symptoms in this region during their lifetime [1].

A study by McClure et al. [13] sought to determine the incidence of metastases in the maxillofacial region at a tertiary care reference center, thus they evaluated 1221 new patients with maxillofacial/oral cancer over 15 years. From these, 26 (16 men and 10 women) were identified as having a histologically confirmed metastasis to the maxillofacial region, at an incidence of 2.1 %. Only one of these patients had cholangiocarcinoma metastasis to the maxillofacial region. Thus, the authors state that any malignant tumor that occurs in the body can metastasize to the maxillofacial region, but some tumors such as lung, breast, and colon do it more often.

There is clinical and investigative evidence that some tumors will metastasize to certain areas of the body and, although the most common metastatic carcinomas in the maxillofacial area are lung and breast, it can also host several malignant diseases from other sources [13]. In the present case, the 33-year-old patient had mandibular metastasis from a primary extrahepatic biliary carcinoma, seeking care when noticing jaw locking, dysphagia and pain, necrosis and drainage in the mental region, and an increased volume on the right side of the mandibular body. The average age for diagnosing cholangiocarcinoma in western countries is 50 years, with the majority found in patients older than 65 years [2]. The present report, therefore, exposes the unusual finding of this type of lesion in a young patient.

Nakanishi et al. [14] reported the case of a 77-year-old patient diagnosed with cholangiocarcinoma who noticed an extremely fast-growing mass involving the lower gum and the entire right floor of the mouth, eventually causing significant trismus. The histopathological examination revealed that this lesion was moderate to slightly differentiated adenocarcinoma compatible with metastatic cholangiocarcinoma. Oral metastases are usually evidence of disseminated disease, as in the case cited, considering that the patient's CT scans also revealed several pulmonary nodules, indicating a severe prognosis.

You et al. [1] reported the case of a 54-year-old patient diagnosed with intrahepatic cholangiocarcinoma presenting metastasis in the thoracic spine and the right posterior body of the mandible. A needle aspiration biopsy of the mandibular mass revealed metastatic adenocarcinoma of the liver. The patient refused the right hemimandibulectomy treatment and opted for radiotherapy, which can be used as a palliative treatment [15]. As the prognosis of metastatic lesions in the mandible is unfavorable, the combination of radio- and chemotherapy to minimize symptoms is also a chosen therapeutic modality, as the case described in the present study.

In almost 30 % of patients with oral metastases, an oral lesion is the first indication of undiscovered malignancy in a distant location. The time from the onset of metastasis to death is usually a few months [1]. Moreover, due to its rarity, the diagnosis of metastatic lesions in the mandible is often late, which may further disfavor the prognosis of this condition. Thus, physicians and dentists must be attentive and aware of the clinical and radiographic characteristics of metastatic tumors. Carefully examining patients with mandibular bone lesions is strongly suggested, especially patients with known malignancy.

## 4 Conclusion

To the best of our knowledge, as of now, there is no case report of jaw metastasis from extrahepatic biliary primary carcinoma in young patients in the literature. As the prognosis of the metastatic lesions to the mandible is very poor, combination of radio and chemotherapy to minimize the symptoms are the chosen therapeutic modality. Because of its rarity, the diagnosis of metastatic lesions in the mandible is very often missed or lately done. So, clinical and histopathological investigations should be done to diagnose the metastatic lesion and its origin.

## Ethical approval

The present study is not a research involving humans, but a clinical case report, whose patient authorised the publication by means of a free and informed consent term.

## Sources of funding

This study had no source of funding.

## Consent

Written informed consent was obtained from the patient for publication of this case report and accompanying images. A copy of the written consent is available for review by the Editor-in-Chief of this journal upon request.

## Author contribution

Letícia Copatti Dogenski – Literature review, translation and spelling revision; conception and design of the study.

Alessandra Kuhn Dall'Magro – Execution of the surgical step; acquisition of data.

Eduardo Dall'Magro – Literature review, translation and spelling revision; conception and design of the study.

Larissa Cunha Cé - Microscopic analysis and image acquisition.

Patrícia Bade – Writing work, discussion and final approval; conception and design of the study.

João Paulo De Carli – Execution of the surgical step; writing work, discussion and final approval; conception and design of the study.

## Registration of research studies

Not applicable.

## Guarantor

João Paulo De Carli.

## Provenance and peer review

Not commissioned, externally peer-reviewed.

## Declaration of competing interest

The publication of this article does not generate any conflicts of interest.
